# Trusted and Secure Wireless Sensor Network Designs and Deployments

**DOI:** 10.3390/s17081787

**Published:** 2017-08-04

**Authors:** Ignacio Bravo, Esther Palomar, Alfredo Gardel, José Luis Lázaro

**Affiliations:** 1 Department of Electronics, University of Alcala, Alcalá de Henares, 28871 Madrid, Spain; alfredo.gardel@uah.es (A.G.); josel.lazaro@uah.es (J.L.L.); 2 School of Computing and Digital Technology Birmingham City University, Birmingham B4 7XG, UK; esther.Palomar@bcu.ac.uk

The deployment of wireless sensor networks (WSNs) is a realistic solution for many markets, such as manufacturing and environment monitoring, military and critical infrastructure monitoring, and, more recently, in energy-efficiency and healthcare sectors, due to their great capabilities in acquiring and transmitting data and processing them for different purposes. Current designs and architectures use radio channel(s) to share information between nodes and a gateway/hub, and implement embedded sensors with autonomous battery or low power microprocessors. Standard platforms, such as Telos B, and operating systems, such as TinyOS or Contiki, are used by a majority of stakeholders.

Security, network topology, and communication protocol are critical issues in the current deployment of WSN applications. Different strategies should be developed according to the application requirements, such as distance, number of transmissions during a period of time, authentication needs, and rate of the frequency band, to name a few.

This Special Issue is aimed at fostering the latest developments in the design, implementation, and evaluation in the field of WSN deployments. 

## Summary of the Special Issue

This Special Issue has focused attention on the research lines related to wireless sensor network (WSN) deployment, such as new contributions to better sensor nodes placement, enhanced routing algorithms, and alternatives to obtaining increased security. 

Sensor placement is an important task not fully studied in WSN applications. The number of sensors and their location will affect the performance, accuracy, and cost of the deployment. Thus, different relevant papers have been published in this particular topic, specifically WSN setups for indoor positioning. Routing is another one of the most noticeable research challenges in WSNs. Large WSNs depend on routing protocol to perform correctly. This Special Issue focuses on papers describing how the path is built by selecting appropriate nodes that are traversed along the network while maintaining a safe/secure communication. Finally, security and authentication in WSNs represent another challenging topic. Nodes in a WSNs can dynamically enter or leave a network, which leads to a variable network topology. Moreover, there are legitimate and eavesdropper nodes operating at the same time in a WSN. The published papers present contributions to balance the different tradeoffs for security and WSN shortcuts providing closed-form equations to quantify and compare the different scenarios, allowing the designer to choose the best alternative.

## Relevant Contributions Related to Sensor Placement 

Sensor placement is an important task in sensor network-based applications such as the design of indoor positioning systems, since the number of sensors and their location affect the accuracy and cost of the whole system. Among the available methods for estimating the position of a target, range-based localization systems use anchor nodes and measurements that can be converted into distances or distance differences; e.g., time of arrival (TOA), time-difference of arrival (TDOA), received signal strength (RSS), etc. Once the information on the distances between the target and the anchor nodes have been obtained, trilateration or multilateration can be used to estimate the position of the target (considering that the target is an emitter and the anchors are sensors). Thus, it is very important that the placement of sensors; in a realistic situation, it is needed for placing sensors to cover the whole area, or not, in order to obtain the best accuracy in certain areas. Placing sensors should minimize the position error bound, which is a widely used metric.

In Reference [1], after reviewing the state-of-the-art of sensor placement for localization, authors have reached the neediness to resort to heuristic methods when deploying sensors in a complex scenario, considering complex functions for the measurement error model or focusing on a whole region of interest (ROI) instead of a single target. In the work, multi-objective evolutionary optimization is applied to obtain the optimum sensor placement. Inspired by works in the literature, the well-known non-dominated sorting genetic algorithm (NSGA-II) has been adapted to solve the sensor placement problem for target localization. The work is a research continuation of previous works from the same authors, where they used a standard multi-objective genetic algorithm to place sensors after considering multiple criteria: placing a fixed number of sensors for localization with range-difference measurements; considering a variable number of sensors, as well as non-line of sight (NLOS) conditions; etc. Applying the algorithm considering a variable number of sensors, without modifications, causes severe problems. Thus, the authors have had to modify the original NSGA-II by adding speciation and evolving subpopulations according to the size of different sensor sets. The obtained results show a considerable improvement over the standard NSGA-II.

The advantages of this work compared to other works in optimum sensor placement for target localization include the multi-objective optimization of different metrics from the Cramér–Rao lower bound; the unconstrained position of the sensors; the consideration of obstacles that can cause occlusions to NLOS sensors; and that the number of sensors can vary within an interval. 

Since conflicting objectives have been optimized, the authors obtained a set of Pareto optimal solutions. This supposes the greatest advantage of multi-objective optimization, since every optimal solution has been obtained quantifying the values of those objectives. This information can be used by the resource manager according to the current needs and availability. Until now, there are not any other researchers that address the sensor placement problem for localization in a similar way. Most of the multi-objective optimization works referenced in surveys focus on sensor deployment for optimizing coverage and energy management, and those that deal with target tracking only address the sensor-scheduling problem.

## Relevant Contributions Related to Routing Algorithms 

In addition to sensor network deployment and location, routing is another one of the most noticeable research challenges in WSN that has gained much ground, due to the emergence of Cyber-Physical Systems (CPS) and the Internet of Things (IoT), which employ sensor devices for communication. Although the sensor has resource constraints in computation, memory, bandwidth, and energy, a routing protocol is required to provide a safe and reliable path to ensure communication. This path is built by selecting appropriate nodes that are traversed from one end of the network to another. However, several security approaches used by routing protocols are inefficient in mitigating attacks. This is because of the high resource consumption of the pertinent mechanisms employed for the security solutions on the sensor node. Therefore, resource-bound security solutions are highly needed to provide minimal active security protection.

Resource-bound security solutions are the process of using improved protocol’s operation semantics to provide adequate security. It can be activated when needed to optimize protocol performance while minimizing security threats using minimal resource consumption. In this situation, it is important to take into account attackers that are able to temporally determine the presence of data in nodes and, importantly, to predict the next forwarding node. Attackers successful in such a siege can further decide to drop all, partially drop, or insert data into the packet. Any of the attacks can drastically degrade the performance of a network to an undesirable level.

*(a)* *Routing Algorithm for Wireless Sensor Networks*

Other important challenges are to identify appropriate forwarding nodes that would mitigate packet loss, a higher performance in packet delivery, as well as provide a better trade-off between security and performance. In Reference [2], a Fuzzy-based Geographic Forwarding protocol (FuGeF) is proposed to improve node selection. The protocol first utilizes a pseudo-random form of dynamism and relies on parameters such as remaining energy, connectivity cost, and progressive distance for node selection. It then employs a Fuzzy Logic System (FLS) for decision-making. The goal of FuGeF is to identify appropriate forwarding nodes that would mitigate packet loss as well as provide a better trade-off between security and base performance. Extensive simulation experiments have been conducted to evaluate the performance of the proposed FuGeF with the Dynamic Window Secured Implicit Geographic Forwarding (DWSIGF) protocol.

The results obtained in Reference [2] show that the FuGeF achieves a higher performance in terms of packet delivery ratio and minimizes the possibility of choosing an attacker as compared to the DWSIGF protocol. In Reference [2], the proposed approach builds on the DWSIGF protocol, leveraging the use of the three abovementioned parameters, as well as an FLS for node selection. These parameters describe features external to a node, as well as influence the distribution of the traffic load among the nodes. The FLS is used to evaluate the chance value of each next hop node based on its three criteria values. The values obtained are used to determine the most appropriate forwarding node for the routing process. Additionally, the dynamism introduced in DWSIGF was maintained with a slight modification such that a shift in the proposed protocol semantics is also achieved. The main aim of the proposed FuGeF is to achieve efficient security while maintaining an acceptable level of base performance.

In an effort to mitigate the effect of attacks on a network, a resource-bound security solution suggests altering the protocol semantics or installing mechanisms that would prevent the selection of a malicious entity as well as maintain the base performance of the protocol.

The authors’ future works lie in strengthening FuGeF in an attempt to thwart more devastating security threats to a network, and to provide comprehensive results, both theoretical and practical.

*(b)* *Dynamic and Adaptive Routing Algorithm *

Smart Cities is one of the many reasons for the huge deployment of WSNs with different goals. For configuring, monitoring, and collecting information from those sensors, low-cost embedded systems provide us a simple platform to be used. However, if the deployment of different WSNs is done in large scenarios such as in a city, hybrid-communication systems (fixed and mobile) must work together.

In Reference [3], a complete review of different wireless protocols such us IEEE 802.15.4 or RPL-6LoWPAN was conducted in order to understand the most common ways to communicate WSNs. The authors of Reference [3] presented a novel proposal called DARAL: A Dynamic and Adaptive Routing Algorithm for Wireless Sensor Networks in order to reduce the control messaged overhead and using energy consumption in the setup stage. This is very useful when low-cost embedded architectures are used to send/receive the information from/to sensors.

The DARAL approach is based on a centralized non-beaconing routing algorithm taking the clustering idea as main feature for a medium-low deployment of sensors, for example in a Smart City. The cluster idea is a good advantage for Smart Cities because different areas of the city will be able to be split into a cluster. Thus, the communication traffic will be reduced due to the main exchange of information that will be conducted in each cluster, minimizing the interferences between nodes from different clusters. A specific set of instructions oriented to minimize the number of communication transactions has been developed to be implemented within the DARAL proposal.

Wireless communication works on IEEE 802.15.4 standard allows the joining of different platforms that support this standard. In addition, one of the benefits of implementing this protocol in each node is the possibility to use well-recognized simulators such as OMNeT++. Thus, the authors have generated multiple scenarios for a Smart City with different initial considerations showing the benefits in terms of energy consumption and data speed.

## Relevant Contributions Related to Security

Finally, in this Special Issue, we have included a section concerning how to secure a WSN. Security and authentication in WSNs face a more challenging environment compared to traditional networks. WSNs have an ad-hoc nature in which the nodes can dynamically enter or leave the network, which leads to a variable network topology. Consequently, there is no predefined route for data replication. With the ambiguity of the nodes involved, a critical problem may occur when a malicious intruder attacks the system. In addition, power limitation can cause the node itself to behave selfishly in order to conserve its energy, which increases the risk of network malfunctioning. Therefore, the abovementioned aspects of the WSNs render security schemes in WSNs more challenging and vulnerable. For this reason, security in WSNs has gained increasing interest.

Game theory is a modern branch of intelligent optimization that tackles problems where the cost functions of different entities are mutually dependent. Game theory has been widely applied to model the behavior in a variety of applications. Recently, with the emergence of infrastructure-less and distributed systems, game theory has found its way into decentralized communication systems. 

*(a)* *Security Requirements and Threads Mitigation*

In several references, cited in Reference [4], multiple techniques have been proposed to meet different WSN security threats mitigation. The security situation, which involves an interaction between the defender(s) and attacker(s), can be directly mapped to a game among players in which each player strives to promote its benefit. More particularly, having the action of the attacker(s) or the defender(s), depending on the counter-action of the other party, places game theory as a perfect fit for this security model. In Reference [4], the authors introduce a brief interpretation of the different game techniques presented in the literature to address WSN security. In addition, an overall view of the desired WSN properties in terms of security fulfillment is presented. This work analyzes game theory-based approaches for the mitigation of different WSN security threats according to state-of-the-art literature on the topic, classifying those approaches into two main categories, namely, cooperative games and non-cooperative games, and each summarizes the involved defense strategies based on game theory. Next, it proposes a taxonomy of game theoretic defense strategies taking into consideration the attacked layer, attack features, attack consequences, convenient defense game approach, and game type. Afterward, a general trust model based on the discussed game theory approaches and scenarios is introduced to take into account the variability and features of the attack types. Consequently, the authors propose the use of this model to any network environment (cooperative/non-cooperative game with internal/external attack). Additionally, they present some applicable future trends for the interested researchers, showing the capability of facing intelligent attacks.

*(b)* *Simulation of Attacks for Security*

One of the keys for the continuous deployment of WSNs is the possibility to use a low-cost embedded platform with a vast support among the developer community. These platforms include sensors to collect information from different scenarios, and this information is usually sent to a central node or other nodes by wireless communication. Nevertheless, the security in this type of platform is one of the values to be analyzed and improved. No specific ways to prevent and avoid attacks are developed by default. Thus, a specific methodology to be implemented in WSNs is developed in Reference [5]. This one will be useful to prevent attacks and give more security to wireless communications.

The new methodology proposes a new way to analyze the attack effects in embedded systems before the final implementation (downloading process) in the microprocessor. Thus, it is possible to check and evaluate the results of different kind of attacks in different scenarios using simulation tools. Only in the case of a success result of the attack in the execution of an ad-hoc software application in the embedded system will it be downloaded.

Taking advantage of the different features of simulation tools, an ideal scenario with different networks, embedded operating systems, or different number of nodes can be evaluated with the methodology proposed in Reference [5]. One interesting issue of this work is the possibility of defining different types of attacks (noise, packets, firmware). This situation is a relevant benefit because different scenarios can be simulated and verified without a real deployment of the network. Virtual platforms have been used to simulate different types of attacks into different networks.

*(c)* *Secure Multiuser Communications*

As has been previously discussed, the spreading use of WSNs requires the increase of security measures and protection/mechanisms to reject attacks or information leakage. This situation is more evident when dealing with multiuser communications. In Reference [6], the authors depict a typical scenario where a multiple antenna base station acts as the router for multiple nodes in the WSN. This router node is a more complex node, which in turn means a higher cost, facilitating the interaction of the WSN nodes with a standard network transmission (e.g., Internet IPv4). The local operation may be tuned as desired. Thus, to secure the multiuser communications in the deployed WSN, a cooperative jammer node has been used. The use of a router and jammer nodes in a WSN deployment may be considered very general and extensible for many applications of current WSNs. The architecture tries to extend the battery lifetime of WSN nodes while providing good secrecy performance. The correct selection of the jammer node increases the performance on secrecy for the WSN.

The proposed secure mechanism in Reference [6] is based on a physical layer security technique, the switch-and-stay combining scheduling scheme. The algorithm requires that the jammer node has the information from a global channel state of both the legitimate channel and the eavesdropper’s channel. Besides, a legitimate channel may be considered as an eavesdropper for a given communication. Two different situations have been analyzed. First, a zero-forcing beamforming scheme is considered to represent a maximum interference from the eavesdropper while avoiding the interruption to the selected legitimate user. Second, the emission of a null-space artificial noise is considered, assuming that the link to the eavesdropper is not known. For both schemas, the achievable secrecy rate has been obtained in closed-form expressions, providing the secrecy outage probability and the effective secrecy throughput, so it is possible to configure the best parameter values for a particular WSN deployment.

*(d)* *Reciprocally-Benefited Secure Transmission *

Cognitive-Radio WSNs are a promising type of network capable of sensing the radio spectrum in its surroundings and of modifying its behavior to improve the overall performance of the WSN. Besides, talking about securing the transmission on the WSN the cognitive-radio approach may be useful. This approach was achieved in Reference [7], where the authors propose a cooperative secure transmission strategy. They consider the existence of a primary standard WSN and a secondary cognitive-radio WSN. The goal is benefit both networks from the cognitive radio sensor network to fully use the available free wireless-spectrum. However, there is a tradeoff between the spectrum sensing accuracy given by the cognitive-radio WSN and the QoS for the standard WSN. In order to further analyze this dependency, the authors study the transmission rate and the primary WSN secrecy rate for a given sensor node to optimally allocate its transmission power considering different threshold conditions. Valuable information related with the power allocation strategy is described with different scenarios to exhibit the performance tradeoff between the transmission rate and the secrecy outage probability. It is worth noting that closed-form expressions are obtained for the transmission outage probability so a merit function can make use of it. The main conclusion is that, to guarantee the secure transmission of the primary WSN, the cognitive-radio should dynamically adjust its transmission power.
